# Leveraging Dental Stem Cells for Oral Health during Pregnancy: A Concise Review

**DOI:** 10.3390/dj12050127

**Published:** 2024-05-07

**Authors:** Aida Meto, Ana Sula, Samuele Peppoloni, Agron Meto, Elisabetta Blasi

**Affiliations:** 1 Department of Dentistry, Faculty of Dental Sciences, University of Aldent, 1007 Tirana, Albania; agron.meto@ual.edu.al; 2 Department of Surgery, Medicine, Dentistry and Morphological Sciences with Interest in Transplant, Oncology and Regenerative Medicine, Laboratory of Microbiology and Virology, University of Modena and Reggio Emilia, 41125 Modena, Italy; samuele.peppoloni@unimore.it (S.P.); elisabetta.blasi@unimore.it (E.B.); 3 Department of Conservative Dentistry and Endodontics, Dr. D.Y. Patil Dental College and Hospital, Dr. D.Y. Patil Vidyapeeth, Pimpri, Pune 411018, Maharashtra, India; 4 Department of Obstetrics and Gynecology, American Hospital, 1060 Tirana, Albania; asula@spitaliamerikan.com

**Keywords:** dental stem cells, maternal-fetal health, pregnancy, hormonal changes, oral microbiota, periodontitis, gingivitis, regenerative dentistry, ethics, safety

## Abstract

Pregnancy induces significant changes in oral health because of hormonal fluctuations, making it a crucial period for preventive measures. Dental stem cells (DSCs), particularly those derived from the dental pulp and periodontal ligaments, offer promising avenues for regenerative therapies and, possibly, preventive interventions. While the use of DSCs already includes various applications in regenerative dentistry in the general population, their use during pregnancy requires careful consideration. This review explores recent advancements, challenges, and prospects in using DSCs to address oral health issues, possibly during pregnancy. Critical aspects of the responsible use of DSCs in pregnant women are discussed, including safety, ethical issues, regulatory frameworks, and the need for interdisciplinary collaborations. We aimed to provide a comprehensive understanding of leveraging DSCs to improve maternal oral health.

## 1. Introduction

In recent years, attention has been focused on oral health during pregnancy [1,2]. Increasing evidence indicates that oral health plays a crucial role in maternal well-being and can affect the health of the developing fetus. Several authors have provided a relevant contribution to the significance of oral health maintenance during pregnancy, offering insights into preventive measures, management strategies, and collaborative efforts necessary among healthcare providers to ensure comprehensive care for expectant mothers [3,4,5]. Pregnancy is a unique stage of a woman’s life, marked by profound physiological changes, including fluctuations in hormone levels, which in turn exert significant effects on oral health [4,6]. These hormonal shifts, particularly elevated levels of estrogen, progesterone, and human chorionic gonadotropin (hCG), establish a setting favorable to oral health changes that, in turn, may favor the occurrence of gingivitis and periodontitis. Indeed, gingivitis, characterized by inflammation of the gums, is highly prevalent during pregnancy, affecting up to 60–75% of pregnant women [3,7]. Moreover, periodontitis, a more severe form of gum disease involving progressive destruction of periodontal tissues, is exacerbated during gestation [3,7,8]. Table 1 provides an overview of oral health indicators and their correlations with pregnancy-related complications. Each indicator is delineated alongside a description of its characteristics and factors contributing to its occurrence. Moreover, the potential risks of pregnancy-related complications associated with each indicator are indicated.

The oral microbiota includes a dynamic and complex ecosystem, including bacteria, fungi, and viruses. Different authors delve into the specific microbial taxa that populate the oral cavity and their potential impact on maternal health during pregnancy [6,7,10,18]. These authors underline how hormonal fluctuations, immune system changes, and alterations in oral hygiene practices can influence the composition and diversity of oral microbiota, potentially leading to dysbiosis. The latter, characterized by disruptions in the balance of microbial communities and heightened pathogenic activity, has a significant impact on pregnancy outcomes. Notably, specific microbial species such as *Porphyromonas gingivalis* and *Fusobacterium nucleatum*, widely recognized for their involvement in periodontal disease, have emerged as relevant players during pregnancy. Indeed, they have been linked to adverse outcomes, particularly preterm birth and low birth weight [19]. Carrouel et al., specifically focused on the oral microbiota of pregnant women [20]. In particular, the presence and relative abundance of periodontal pathogens was assessed within the interdental microbiota of pregnant individuals with an intact periodontium, at 3 months of gestation. Through molecular and culture-based assays, several microbial species, including *P. gingivalis*, *Tannerella forsythia*, and *Treponema denticola*, among others, were detected. Given the well-known role of these pathogens in periodontal diseases, the authors highlight the potential implications of the presence of these microbial communities on maternal oral health during pregnancy and the potential risk of subsequent periodontal diseases.

Recently, the association between oral microbiota and hypertensive disorders during pregnancy, particularly preeclampsia, has become a focal point of investigation [21]. Preeclampsia, characterized by high blood pressure and organ dysfunction during pregnancy, represents a major health concern. The significance of maternal oral health extends beyond this scope, as emerging evidence suggests a potential link between periodontal diseases and several adverse pregnancy outcomes, including preterm birth and low birth weight [8,10,19,22]. This bidirectional relationship underscores the importance of proactive oral care interventions during pregnancy to safeguard maternal and fetal well-being. Maintaining optimal oral health during pregnancy is crucial for both maternal and fetal well-being. Nevertheless, there is considerable variability in the awareness and adherence to recommended oral hygiene practices among expectant mothers. In their study, Bushehab et al., offered valuable insights into the impact of oral hygiene practices and awareness levels of pregnant women [3]. Employing methodologies such as surveys, interviews, or questionnaires, these authors gathered data to assess the knowledge, attitudes, and behaviors of pregnant women concerning oral health during pregnancy and its potential implications for pregnancy outcomes.

Given the evolving landscape of oral health and the dynamic physiological shifts that occur during pregnancy, there is growing interest in investigating minimally invasive approaches to manage various oral clinical conditions in pregnant women, with a focus on ensuring their comfort and well-being.

With an understanding of the pivotal roles of stem cells in both medicine and dentistry, this concise review considers the potential use of dental stem cells (DSCs) throughout pregnancy across diverse clinical contexts. The overarching goal is to mitigate concerns regarding fear, pain, trauma, and fetal risks associated with interventions. Despite the inherent challenges stemming from limited research in this domain, this initiative is poised to assist research entities in refining strategies for future employment of DSCs to enhance oral health outcomes in expectant mothers.

## 2. DSCs and Pregnancy

### 2.1. A Brief History of Stem Cells

A synthetic journey through history provides invaluable insights into the significance of DSCs, beginning with the discovery of stem cells themselves. Stem cells have long captivated scientists due to their extraordinary regenerative potential, with roots tracing back to Ernst Haeckel’s proposal of “Stammzellen” in 1868 [23]. The late 20th century witnessed pivotal moments in stem cell research, including Alexander Maksimov’s coining of the term “stem cell” in 1908 and the groundbreaking isolation of embryonic stem cells (ESCs) from mouse embryos in 1981 [24]. A monumental leap occurred in 1998 when James Thomson and his team successfully isolated human ESCs, marking a significant milestone [25]. Subsequently, in 2006, Shinya Yamanaka’s discovery of induced pluripotent stem cells (iPSCs) revolutionized the field by providing a method to reprogram adult cells into a pluripotent state, offering an ethically acceptable alternative to ESCs [26]. This breakthrough offered a priceless alternative to ESCs by circumventing the need for human embryos. Stem cell research expanded to encompass various types of adult stem cells, including mesenchymal stem cells (MSCs) found in bone marrow and adipose tissue, among other sources [27]. Next, DSCs emerged as promising candidates, when sources such as dental pulp, periodontal ligaments, and dental follicles provided an easily accessible reservoir of cells for future applications in regenerative therapies [28,29,30,31]. Through the exploration of stem cell origins and historical milestones, researchers have paved the way for advancements in regenerative medicine, offering potential solutions for treating a myriad of diseases and injuries.

### 2.2. What Do We Know about DSCs?

DSCs represent a valuable asset with vast therapeutic potential, not only within dentistry but also extending to a broader range of medical applications [28,30,31]. Undoubtedly, to fully harness their benefits, further research efforts are imperative to unravel their intricate mechanisms of action, refine isolation and culture methodologies, and bridge the gap between preclinical discoveries and clinical implementation. Delving into the complexity of DSCs unveils their unique characteristics, various sources, intrinsic properties, and promising applications. For researchers, these aspects represent novel pathways that can be utilized in both regenerative medicine and oral healthcare.

Table 2 provides a concise overview of various types of dental stem cells, including their sources, functions, and potential applications in regenerative medicine and oral healthcare. DSCs, derived from different tissues such as dental pulp, periodontal ligaments, and oral mucosa, exhibit unique properties such as self-renewal and multilineage differentiation potential. These cells hold significant promise for tissue regeneration and repair in dentistry, offering potential treatments for a range of oral and systemic diseases, including periodontal disease, dental caries, and craniofacial defects.

### 2.3. Isolation of DSCs: Standardization of Protocols

The standardization of experimental protocols aimed at obtaining DSCs is essential for significant advancement in basic and clinical research. Table 3 provides a summary of the published protocols for the in vitro isolation of various types of stem cells from different anatomical niches in the oral cavity. For each stem cell type, step-by-step procedures are described, including tissue collection, dissociation, cell isolation, and culture [48,49,50,51,52,53]. Strict adherence to these standardized isolation protocols is essential and will undoubtedly enhance the reproducibility and scalability of stem cell-based therapies and research efforts in regenerative dentistry and beyond.

## 3. Clinical Implications and Applications of DSCs in Oral Health

Minimizing oral disease-related complications during pregnancy remains a widely recognized key point to be pursued. Monitoring oral health indicators during prenatal care allows for early intervention, potentially mitigating the risk of pregnancy-related complications [56]. Educating pregnant women about the importance of oral hygiene practices and regular dental check-ups is crucial in preventing potential complications [57]. Implementing comprehensive oral health education programs can empower women to adopt preventive measures and enhance their overall well-being during pregnancy [58]. In this scenario, the clinical applications of DSCs during pregnancy represent a novel and highly challenging tool to work with. The following are some of the most likely possibilities for DSCs’ use in pregnant women:

1. *Prevention and Treatment of Dental Caries*: DSCs can be employed for the development of novel strategies for the remineralization of dental enamel and dentin, offering a non-invasive approach to prevent and treat dental caries in pregnant women [59].
2. *Pulp Regeneration:* Pulpal diseases, including pulpitis and pulp necrosis, can pose significant challenges during pregnancy due to limited treatment options that are safe for both the mother and fetus [34]. DSC-mediated pulp regeneration involves the transplantation of DSCs, such as stem cells from the apical papilla (SCAPs) or DPSCs, into the pulp chamber to promote pulp tissue regeneration and repair [30,31,32]. This approach holds promise for preserving the vitality of compromised teeth and avoiding invasive procedures during pregnancy.
3. *Periodontal Disease Management*: Preclinical studies have demonstrated the efficacy of DSC-based therapies in promoting periodontal tissue regeneration and reducing inflammation, offering a potential treatment modality for pregnant women with periodontal disease [37,52,56,60].
4. *Salivary Gland Regeneration*: DSC-based approaches for salivary gland regeneration offer a potential solution for restoring salivary gland function and alleviating xerostomia in pregnant women [61,62].
5. *Oral-Origin Organoid Transplantation*: Recent advancements in stem cell research have led to the development of oral-origin organoids, including tooth germ organoids, salivary gland organoids, taste bud organoids, and lingual epithelial organoids [40,42]. These organoids hold promise for regenerative therapies in maternal oral health enhancement. Transplantation of organoids derived from oral tissues may offer innovative approaches for repairing damaged oral structures and restoring oral function during pregnancy.
6. *Neural Regeneration*: Pregnancy-related neuropathies and nerve injuries in the oral and maxillofacial region can lead to significant discomfort and functional impairment. DSCs have shown promise in promoting neural regeneration and may offer novel therapeutic avenues for managing pregnancy-related neuropathic pain and sensory disturbances [35,45,63].

These clinical implications underscore the potential of DSCs as versatile tools for enhancing maternal oral health during pregnancy. Further research and clinical studies are needed to validate the safety and efficacy of DSC-based therapies in pregnant women.

## 4. Limitations for DSC Use in Pregnancy

Currently, no studies specifically address the use of DSCs to counteract oral lesions during pregnancy. The most likely explanations might be the potential risks and ethical considerations associated with such interventions, including the ones indicated below.

*Safety Concerns*: Pregnancy is a sensitive period during which any intervention, including the use of stem cells, may pose risks to the mother and developing fetus. Stem cell therapies carry inherent risks such as immune rejection, infection, and tumorigenicity [56,64]. Additionally, the effects of stem cell therapy on fetal development are not well understood, raising concerns about potential adverse outcomes.

*Ethical Considerations*: Conducting research involving pregnant women raises complex ethical issues, including the need to ensure the safety and well-being of both the mother and fetus [65]. Researchers must adhere to strict ethical guidelines and obtain informed consent from participants, considering the potential risks and benefits of the intervention.

*Regulatory Hurdles*: Stem cell therapies, particularly those involving novel applications, such as during pregnancy, may face regulatory challenges and require extensive preclinical and clinical testing to ensure safety and efficacy [66]. The lack of established protocols and regulatory frameworks for using stem cells during pregnancy may hinder research in this area [67].

*Limited Evidence*: Despite the potential therapeutic benefits of stem cell therapy, including DSC treatment of oral lesions, there is still limited evidence of their safety and efficacy, specifically in pregnant women [68]. The absence of robust clinical data may deter researchers and clinicians from further exploring this application.

Table 4 shows the critical points currently affecting DSCs’ employment in pregnancy. Overcoming such limitations through further research, interdisciplinary collaboration, long-term monitoring, and adherence to ethical and regulatory standards will enhance the credibility and applicability of leveraging DSCs in pregnancy. Overall, leveraging DSCs during pregnancy to enhance oral health remains an objective to be pursued.

## 5. Future Trajectories of DSCs during Pregnancy

**Scheme 1:**
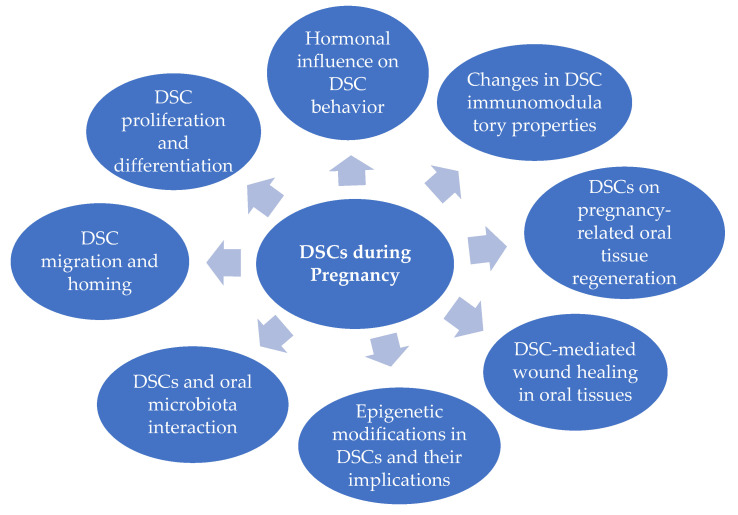
Potential impact of pregnancy on DSCs. Different lines of research will have to be developed to expand the knowledge on each specific issue.

Extensive research should focus on the therapeutic potential of DSCs in pregnancy-associated oral diseases, particularly elucidating the biomolecular mechanisms responsible for the beneficial effects. Furthermore, efforts should be directed toward optimizing the delivery methods and dosing regimens of DSC-based therapies to maximize their therapeutic efficacy. Scheme 1 outlines key issues that may deserve special attention and should ideally become priority topics for assessment by both basic and clinical researchers. The ability of DSCs to proliferate and differentiate during pregnancy (even at different stages of fetal development), their capacity to migrate and regulate immune responses in response to hormonal changes, and the potential alterations in their interaction with the oral cavity microbiota influenced by pregnancy are all crucial aspects that need clarification. A deep understanding of these issues will promote the clinical use of DSCs during pregnancy, especially regarding their role in maternal oral healing, tissue regeneration, and fetal wellness.

## 6. Conclusions

DSCs stand as a promising source of multipotent cells with substantial potential for advancing regenerative dentistry and oral health enhancement. These cells can be isolated from various oral tissues, primarily including the dental pulp, periodontal ligament, and dental follicles. DSCs possess notable versatility, demonstrating the ability to differentiate into multiple cell lineages, such as osteoblasts, odontoblasts, adipocytes, and neural cells. This adaptability makes DSCs attractive candidates for diverse clinical applications, ranging from tissue engineering to interventions addressing pregnancy-related oral health challenges. Despite significant progress in DSC research, numerous challenges persist. These encompass the need for standardization in isolation protocols, the enhancement of cell proliferation and differentiation, the optimization of scaffold design, rigorous evaluation of safety and efficacy, and the attainment of regulatory approval for clinical implementation. Overcoming these obstacles is crucial to fully harness the therapeutic potential of DSC-based therapies and propel the field of regenerative dentistry forward. In this pursuit, establishing interdisciplinary collaborations among healthcare practitioners is pivotal. Such collaborative efforts will drive further exploration in basic and preclinical research, facilitating the seamless integration of DSC therapies into specialized clinical contexts, including those relevant to pregnancy and prenatal care.

## Figures and Tables

**Table 1:** Correlation between oral health indicators and pregnancy-related complications.

Oral Health Indicator	Description	Factors	Potential Risks of Pregnancy-Related Complications	References
Pregnancy Gingivitis	Swelling, redness, sensitive, or painful gingiva	Hormonal changes, poor oral hygiene, bacterial plaque accumulation	Increased risk of preterm birth, low birth weight	[3,6,7,8,9,10]
Periodontal Disease	Inflammation and infection of the gums and surrounding tissues	Hormonal changes, bacterial plaque accumulation, smoking, genetic predisposition	Linked to preterm birth, preeclampsia, gestational diabetes	[3,6,7,8,9,10]
Dental Caries	Tooth decay resulting from bacterial acid demineralization	Poor oral hygiene, dietary factors, bacterial plaque accumulation	Enhanced risk of preterm birth, low birth weight	[10,11]
Oral Candidiasis	Fungal infection characterized by white patches in the mouth	Hormonal changes, immunosuppression, poor oral hygiene	Potential risk factor for preterm birth	[12]
Oral Lesions	Abnormalities in the oral mucosa, including ulcers, red or white patches	Nutritional deficiencies, hormonal changes, viral infections	Linked to maternal health complications and fetal development	[13,14]
Pregnancy Tumors	Also known as pyogenic granulomas or granuloma gravidarum	Hormonal imbalances, poor oral hygiene, local irritants	Typically resolve postpartum	[15,16]
Salivary Changes	Altered salivary composition, dry mouth	Hormonal changes, dehydration, medication side effects	May contribute to gestational diabetes	[17]

**Table 2:** Comprehensive overview of the different DSCs, listed according to their characteristics, sources, functions, and potential applications.

Characteristics	Sources	Functions	Promising Applications	References
DSCs are found within various dental tissues, including dental pulp, periodontal ligaments, dental follicles, and the dental papilla. DSCs possess self-renewal capabilities and multilineage differentiation potential into osteoblasts, odontoblasts, adipocytes, chondrocytes, and neural-like cells. DSCs express specific cell surface markers such as STRO-1, CD146, CD90, CD73, and CD105, facilitating their isolation and identification.	Dental pulp stem cells (DPSCs) are derived from the dental pulp tissue within the pulp chamber of teeth.	DSCs exhibit immunomodulatory and anti-inflammatory effects, regulating immune responses and mitigating inflammation associated with oral diseases. DSCs secrete bioactive molecules, growth factors, and cytokines promoting tissue regeneration, angiogenesis, and wound healing. DSCs demonstrate plasticity and adaptability, responding to microenvironmental cues and differentiating into specialized cell types for tissue repair and regeneration.	DSCs hold promise for regenerative medicine applications, including dental tissue, bone, and nerve regeneration. They are being investigated for the treatment of various oral and systemic diseases such as periodontal disease, dental caries, pulpitis, and craniofacial defects. DSC-based therapies show promising results in preclinical studies and animal models, indicating potential for future clinical translation.	[28,29,30,31,32,33,34,35]
	Periodontal ligament stem cells (PDLSCs) are found within the periodontal ligament that surrounds and attaches teeth to the alveolar bone.			
	Dental follicle stem cells (DFSCs) are located within the dental follicle, a loose connective tissue surrounding the developing tooth germ.			
	Dental papilla stem cells (DPaSCs) reside within the dental papilla, a soft tissue core of the developing tooth.			
Stem Cells from Human Exfoliated Deciduous Teeth (SHEDs) are derived from the dental pulp of deciduous teeth that have been naturally shed or extracted. These stem cells possess unique characteristics, including high proliferation rates, multipotent differentiation capabilities, and robust regenerative potential. SHEDs exhibit a spindle-shaped morphology and express specific cell surface markers associated with MSCs, such as CD73, CD90, and CD105.	SHEDs are sourced from the dental pulp tissue of deciduous teeth, which are typically collected following natural exfoliation or extraction of primary teeth during childhood. The dental pulp is isolated from the teeth and processed to obtain a heterogeneous population of SHEDs, which can be cultured and expanded in vitro for various research and therapeutic purposes.	SHEDs possess several unique properties that make them attractive for regenerative medicine applications. These include their high proliferation rates, which enable rapid expansion in culture, and their multipotent differentiation capabilities, allowing them to differentiate into various cell types, including odontoblasts, adipocytes, and neural cells. Additionally, SHEDs secrete a range of bioactive molecules and growth factors that contribute to tissue repair, angiogenesis, and immunomodulation.	SHEDs can differentiate into odontoblast-like cells and contribute to the regeneration of dentin and pulp tissues damaged by dental caries, trauma, or disease. SHEDs may be used in conjunction with biomaterial scaffolds to regenerate bone and soft tissue defects in the craniofacial region resulting from congenital anomalies, trauma, or surgical interventions. SHEDs have the potential to differentiate into neural-like cells and promote nerve regeneration in the oral and maxillofacial region following nerve injury or damage.	[33,36]
Oral Mucosa Stem Cells (OMSCs) are a type of epithelial stem cell found in the oral mucosa, which lines the inside of the mouth and covers the gums, inner cheeks, lips, tongue, and the roof of the mouth (palate). These stem cells exhibit self-renewal capacity and multipotent differentiation potential, enabling them to give rise to various cell types within the epithelial lineage, such as keratinocytes and other epithelial cell types.	OMSCs are primarily sourced from the oral mucosa tissue, which can be easily accessed through minimally invasive procedures such as biopsy. Additionally, OMSCs can be isolated from oral mucosa scrapings or discarded tissues obtained during routine dental procedures, making them a readily available cell source for research and regenerative medicine applications.	OMSCs possess several key properties that make them attractive for tissue regeneration and repair in the oral cavity. OMSCs also exhibit barrier function, immunomodulatory effects, and angiogenic potential, allowing them to protect against pathogens, modulate immune responses, and promote tissue vascularization and wound healing in the oral mucosa.	Treatment of oral mucosal injuries and wounds, such as ulcers, lacerations, and burns. Regeneration of oral mucosal tissues damaged by trauma, disease, or surgical procedures. Reconstruction of oral mucosal defects resulting from congenital anomalies or oncologic resections. Engineering of functional oral mucosal grafts for use in tissue grafting and transplantation procedures.	[37,38,39]
Lingual epithelial stem cells (LESCs) are a specialized population of epithelial stem cells found within the lingual epithelium of the tongue. These stem cells possess unique characteristics, including their ability to self-renew and differentiate into various cell types within the epithelial lineage. LESCs play a crucial role in maintaining the homeostasis and integrity of the lingual epithelium, which undergoes constant renewal to replenish lost or damaged cells.	LESCs are primarily sourced from the lingual epithelium, which is the mucous membrane covering the dorsal surface of the tongue. Within the lingual epithelium, LESCs reside in specialized niches known as taste buds and interfollicular epithelium. These stem cells can be isolated from lingual tissue biopsies or obtained through non-invasive sampling methods such as brush biopsies.	LESCs exhibit properties characteristic of epithelial stem cells, including their capacity for self-renewal and multipotent differentiation. These stem cells undergo asymmetric division to generate daughter cells with distinct fates: one retains stem cell properties while the other undergoes differentiation into specialized cell types, such as keratinocytes, taste bud cells, and other epithelial cell types.	LESCs can be utilized to regenerate damaged or diseased lingual epithelium, such as in cases of oral mucosal injuries, ulcers, or epithelial defects resulting from trauma or pathology. Given their location within taste buds, LESCs may contribute to the regeneration of taste buds lost due to aging, injury, or disease. These stem cells could serve as a cell source for generating bioengineered tissues that closely mimic the structure and function of native lingual epithelium.	[40,41,42]
Gingival mesenchymal stem cells (GMSCs) are a subpopulation of MSCs residing within the gingival tissue, which is part of the oral mucosa. These stem cells possess distinctive characteristics, including their ability to self-renew and differentiate into various cell lineages of mesenchymal origin. GMSCs play a crucial role in maintaining the homeostasis and integrity of the gingival tissue, contributing to its regenerative capacity and response to injury or inflammation.	GMSCs are primarily sourced from the gingival tissue, which comprises the soft tissue surrounding the teeth and underlying alveolar bone. Within the gingival tissue, GMSCs reside in the periodontal ligament, gingival connective tissue, and gingival epithelium. These stem cells can be isolated from gingival tissue biopsies obtained during routine dental procedures or periodontal surgery.	GMSCs possess a high proliferation rate and multilineage differentiation potential, enabling them to differentiate into various cell types such as osteoblasts, adipocytes, chondrocytes, and fibroblasts. These stem cells also secrete a range of bioactive molecules, growth factors, and cytokines that modulate the local microenvironment and facilitate tissue repair and regeneration.	GMSCs can be utilized in regenerative therapies aimed at restoring periodontal tissues damaged by periodontal disease or trauma. These stem cells may promote the regeneration of periodontal ligaments, cementum, and alveolar bone, leading to improved periodontal health and function. GMSCs have been investigated for their role in enhancing the osseointegration of dental implants and promoting bone formation around implant sites. Coating dental implants with GMSC-derived extracellular matrix or seeding implants with GMSCs may improve implant stability and long-term success rates. GMSCs may be employed in the treatment of oral mucosal disorders such as oral lichen planus, oral ulcers, and mucosal defects resulting from trauma or surgical procedures.	[43,44,45,46,47]

**Table 3:** Protocols available in the literature for stem cell isolation from the oral cavity.

Stem Cell Type	Isolation Protocol
DPSCs [48,49,50,51]	1. Tooth extraction: Obtain extracted teeth, preferably third molars, from patients. 2. Pulp tissue isolation: Sterilize the tooth surface and crack open the tooth to access the pulp chamber. 3. Pulp tissue dissociation: Digest the pulp tissue using enzymatic solutions such as collagenase and dispase. 4. Cell isolation: Centrifuge the digested pulp tissue to obtain a cell pellet, then culture the isolated cells in appropriate growth media.
PDLSCs [51,52]	1. Gingival tissue collection: Perform gingival biopsies or extract teeth with surrounding periodontal ligament tissues. 2. Tissue dissociation: Chop the collected gingival tissue into small fragments and digest them enzymatically. 3. Cell isolation: Filter the digested tissue to remove debris, then culture the isolated cells in suitable culture media.
DFSCs [48,49,50,51]	1. Tooth extraction: Obtain developing teeth or extracted teeth containing dental follicles. 2. Follicle isolation: Carefully dissect the dental follicle from the tooth using microsurgical techniques. 3. Tissue dissociation: Chop the isolated dental follicle tissue into small pieces and digest it with collagenase. 4. Cell isolation: Centrifuge the digested tissue, collect the cell pellet, and culture the cells in appropriate media.
DPaSCs [32,54,55]	1. Tooth germ collection: Collect developing tooth germs from embryonic or fetal tissues. 2. Papilla isolation: Dissect the dental papilla tissue from the tooth germ using fine forceps and scalpels. 3. Tissue dissociation: Minutely chop the isolated dental papilla tissue and enzymatically digest it. 4. Cell isolation: Filter the digested tissue, centrifuge the suspension, and culture the isolated cells in suitable culture media.
SHEDs [33,36]	1. Deciduous tooth collection: Obtain naturally exfoliated or extracted deciduous teeth from pediatric patients. 2. Pulp tissue isolation: Sterilize the tooth surface and carefully extract the dental pulp tissue. 3. Tissue dissociation: Mince the extracted pulp tissue and digest it with enzymes like collagenase. 4. Cell isolation: Centrifuge the digested tissue, collect the cell pellet, and culture the cells in appropriate media.
OMSCs [37,38,39]	1. Oral mucosa biopsy: Perform a minimally invasive biopsy procedure to collect oral mucosal tissue samples. 2. Tissue dissociation: Chop the collected tissue samples into small pieces and enzymatically digest them. 3. Cell isolation: Filter the digested tissue, centrifuge the suspension, and culture the isolated cells in suitable media.
LESCs [40,41,42]	1. Lingual tissue collection: Obtain lingual tissue samples from the dorsal surface of the tongue using biopsy or brush biopsy techniques. 2. Epithelial cell enrichment: Separate epithelial cells from the collected tissue using enzymatic or mechanical methods. 3. Stem cell isolation: Identify and isolate lingual epithelial stem cells based on specific markers using flow cytometry or magnetic cell sorting. 4. Cell culture: Culture the isolated stem cells in appropriate media supplemented with growth factors.
GMSCs [53]	1. Gingival tissue collection: Perform gingival biopsies or obtain gingival tissue samples from periodontal surgeries. 2. Tissue dissociation: Chop the collected tissue into small fragments and enzymatically digest them. 3. Cell isolation: Filter the digested tissue, centrifuge the suspension, and culture the isolated cells in suitable media.

**Table 4:** Parameters that may influence the research development in the DSC field.

Parameters	Limitation/Comment
Clinical Evidence	The current body of clinical evidence regarding the use of DSCs in pregnancy-related oral health interventions is limited, potentially hindering the comprehensive assessment of their efficacy and safety in this specific context.
Ethical Considerations	Ethical concerns surrounding the use of stem cells, particularly during pregnancy, may pose challenges in conducting extensive research and implementing therapeutic strategies. Ethical considerations regarding fetal safety, consent procedures, and regulatory frameworks need to be carefully addressed.
Long-term Follow-up	Long-term follow-up data on pregnant individuals who have undergone DSC-based interventions may be lacking, making it difficult to assess the sustained effectiveness and potential adverse effects of these treatments over time.
Study Designs	Variability in study designs, including differences in sample sizes, methodologies, and outcome measures among existing studies, may limit the comparability and generalizability of findings, thus impacting the overall reliability of conclusions drawn from the literature.
Interdisciplinary Collaboration	Given the multifaceted nature of pregnancy-related oral health and the utilization of DSCs, an interdisciplinary collaboration among dental professionals, obstetricians, and researchers is essential. Intense collaboration across disciplines may facilitate the comprehensive understanding and implementation of dental stem cell-based approaches in pregnancy care.
Regulatory Constraints	Regulatory constraints and legal frameworks governing the use of stem cells, particularly in pregnant women, may pose logistical challenges and barriers to the translation of research findings into clinical practice. Compliance with regulatory standards and ethical guidelines is imperative, although it may affect the progress of therapeutic developments.

## References

[1] Hartnett E., Haber J., Krainovich-Miller B., Bella A., Vasilyeva A., Lange Kessler J. (2016). Oral Health in Pregnancy. J. Obstet. Gynecol. Neonatal Nurs..

[2] Pecci-Lloret M.P., Linares-Pérez C., Pecci-Lloret M.R., Rodríguez-Lozano F.J., Oñate-Sánchez R.E. (2024). Oral Manifestations in Pregnant Women: A Systematic Review. J. Clin. Med..

[3] Bushehab N.M.E., Sreedharan J., Reddy S., D’souza J., Abdelmagyd H. (2022). Oral Hygiene Practices and Awareness of Pregnant Women about the Effects of Periodontal Disease on Pregnancy Outcomes. Int. J. Dent..

[4] Chawłowska E., Karasiewicz M., Lipiak A., Staszewski R., Cofta M., Biskupska M., Giernaś B., Zawiejska A. (2022). Oral Health Behaviours, Knowledge, and Literacy of Expectant Mothers: A Cross-Sectional Study among Maternity Ward Patients. Int. J. Environ. Res. Public Health.

[5] Przeklasa-Bierowiec A., Jakubik A., Szczeklik K., Majewska I., Marcinek A., Pytko-Polończyk J. (2020). Awareness of oral health prophylaxis in pregnant women. Folia Med. Crac..

[6] Xu B., Han Y.W. (2022). Oral bacteria, oral health, and adverse pregnancy outcomes. Periodontol 2000.

[7] Balan P., Chong Y.S., Umashankar S., Swarup S., Loke W.M., Lopez V., He H.G., Seneviratne C.J. (2018). Keystone Species in Pregnancy Gingivitis: A Snapshot of Oral Microbiome during Pregnancy and Postpartum Period. Front. Microbiol..

[8] Boggess K.A. (2020). Choosing the left fork: Steven Offenbacher and understanding maternal periodontal disease and adverse pregnancy outcomes. J. Periodontol..

[9] Offenbacher S., Lieff S., Boggess K.A., Murtha A.P., Madianos P.N., Champagne C.M.E., McKaig R.G., Jared H.L., Mauriello S.M., Auten R.L. (2001). Maternal periodontitis and prematurity. Part I: Obstetric outcome of prematurity and growth restriction. Ann. Periodontol..

[10] Vidmar Šimic M., Maver A., Zimani A.N., Hočevar K., Peterlin B., Kovanda A., Premru-Sršen T. (2023). Oral microbiome and preterm birth. Front. Med..

[11] Cho G.J., Kim S.Y., Lee H.C., Kim H.Y., Lee K.M., Han S.W., Oh M.J. (2020). Association between dental caries and adverse pregnancy outcomes. Sci. Rep..

[12] Xiao J., Fogarty C., Wu T.T., Alkhers N., Zeng Y., Thomas M., Youssef M., Wang L., Cowen L., Abdelsalam H. (2019). Oral health and Candida carriage in socioeconomically disadvantaged US pregnant women. BMC Pregnancy Childbirth.

[13] Duarte da Silva K., Vargas-Ferreira F., Dâmaso Bertoldi A., Celso Lopes Fernandes de Barros F., Fernando Demarco F., Britto Correa M., Beatriz Chaves Tarquinio S. (2022). Oral mucosal lesions in pregnant women: A population-based study. Oral Dis..

[14] Bett J.V.S., Batistella E.Â., Melo G., Munhoz E.A., Silva C.A.B., Guerra E.N.D.S., Porporatti A.L., De Luca Canto G. (2019). Prevalence of oral mucosal disorders during pregnancy: A systematic review and meta-analysis. J. Oral Pathol. Med..

[15] Cardoso J.A., Spanemberg J.C., Cherubini K., Figueiredo M.A., Salum F.G. (2013). Oral granuloma gravidarum: A retrospective study of 41 cases in Southern Brazil. J. Appl. Oral Sci..

[16] Lomeli Martinez S.M., Carrillo Contreras N.G., Gómez Sandoval J.R., Zepeda Nuño J.S., Gomez Mireles J.C., Varela Hernández J.J., Mercado-González A.E., Bayardo González R.A., Gutiérrez-Maldonado A.F. (2023). Oral Pyogenic Granuloma: A Narrative Review. Int. J. Mol. Sci..

[17] Crusell M.K.W., Brink L.R., Nielsen T., Allin K.H., Hansen T., Damm P., Lauenborg J., Hansen T.H., Pedersen O. (2020). Gestational diabetes and the human salivary microbiota: A longitudinal study during pregnancy and postpartum. BMC Pregnancy Childbirth.

[18] Saadaoui M., Singh P., Al Khodor S. (2021). Oral microbiome and pregnancy: A bidirectional relationship. J. Reprod. Immunol..

[19] Han Y.W., Redline R.W., Li M., Yin L., Hill G.B., McCormick T.S. (2004). *Fusobacterium nucleatum* induces premature and term stillbirths in pregnant mice: Implication of oral bacteria in preterm birth. Infect. Immun..

[20] Carrouel F., Kanoute A., Lvovschi V.E., Bourgeois D. (2023). Periodontal pathogens of the interdental microbiota in a 3 months pregnant population with an intact periodontium. Front. Microbiol..

[21] Beckers K.F., Sones J.L. (2020). Maternal microbiome and the hypertensive disorder of pregnancy, preeclampsia. Am. J. Physiol. Heart Circ. Physiol..

[22] Ye C., Kapila Y. (2021). Oral microbiome shifts during pregnancy and adverse pregnancy outcomes: Hormonal and Immunologic changes at play. Periodontol 2000.

[23] Armstrong L., Lako M., Buckley N., Lappin T.R., Murphy M.J., Nolta J.A., Pittenger M., Stojkovic M. (2012). Editorial: Our top 10 developments in stem cell biology over the last 30 years. Stem Cells.

[24] Evans M.J., Kaufman M.H. (1981). Establishment in culture of pluripotential cells from mouse embryos. Nature.

[25] Thomson J.A., Itskovitz-Eldor J., Shapiro S.S., Waknitz M.A., Swiergiel J.J., Marshall V.S., Jones J.M. (1998). Embryonic stem cell lines derived from human blastocysts. Science.

[26] Takahashi K., Yamanaka S. (2006). Induction of pluripotent stem cells from mouse embryonic and adult fibroblast cultures by defined factors. Cell.

[27] Pittenger M.F., Mackay A.M., Beck S.C., Jaiswal R.K., Douglas R., Mosca J.D., Moorman M.A., Simonetti D.W., Craig S., Marshak D.R. (1999). Multilineage potential of adult human mesenchymal stem cells. Science.

[28] Tatullo M., Marrelli M., Shakesheff K.M., White L.J. (2015). Dental pulp stem cells: Function, isolation and applications in regenerative medicine. J. Tissue Eng. Regen. Med..

[29] Sun L., Du X., Kuang H., Sun H., Luo W., Yang C. (2023). Stem cell-based therapy in periodontal regeneration: A systematic review and meta-analysis of clinical studies. BMC Oral Health.

[30] Zhang W., Yelick P.C. (2021). Tooth Repair and Regeneration: Potential of Dental Stem Cells. Trends Mol. Med..

[31] Botelho J., Cavacas M.A., Machado V., Mendes J.J. (2017). Dental stem cells: Recent progresses in tissue engineering and regenerative medicine. Ann. Med..

[32] Nagata M., Ono N., Ono W. (2021). Unveiling diversity of stem cells in dental pulp and apical papilla using mouse genetic models: A literature review. Cell Tissue Res..

[33] Shi X., Mao J., Liu Y. (2020). Pulp stem cells derived from human permanent and deciduous teeth: Biological characteristics and therapeutic applications. Stem Cells Transl. Med..

[34] Nakashima M., Iohara K., Murakami M., Nakamura H., Sato Y., Ariji Y., Matsushita K. (2017). Pulp regeneration by transplantation of dental pulp stem cells in pulpitis: A pilot clinical study. Stem Cell Res. Ther..

[35] Vaswani B.K., Mundada B.P., Bhola N., Paul P., Reche A., Ahuja K.P. (2023). Stem-Cell Therapy: Filling Gaps in Oro-Maxillofacial Region. Cureus.

[36] Miura M., Gronthos S., Zhao M., Lu B., Fisher L.W., Robey P.G., Shi S. (2003). SHED: Stem cells from human exfoliated deciduous teeth. Proc. Natl. Acad. Sci. USA.

[37] Wang J., Zhao Z., Yang K., Bai Y. (2024). Research progress in cell therapy for oral diseases: Focus on cell sources and strategies to optimize cell function. Front. Bioeng. Biotechnol..

[38] López S., Hoz L., Tenorio E.P., Buentello B., Magaña F.S., Wintergerst A., Navas A., Garfias Y., Arzate H. (2021). Can Human Oral Mucosa Stem Cells Differentiate to Corneal Epithelia?. Int. J. Mol. Sci..

[39] Calenic B., Greabu M., Caruntu C., Tanase C., Battino M. (2015). Oral keratinocyte stem/progenitor cells: Specific markers, molecular signaling pathways and potential uses. Periodontology 2000.

[40] Hisha H., Tanaka T., Ueno H. (2016). Lingual Epithelial Stem Cells and Organoid Culture of Them. Int. J. Mol. Sci..

[41] Barlow L.A. (2022). The sense of taste: Development, regeneration, and dysfunction. WIREs Mech. Dis..

[42] Barlow L.A. (2015). Progress and renewal in gustation: New insights into taste bud development. Development.

[43] Tolouei A.E., Oruji F., Tehrani S., Rezaei S., Mozaffari A., Jahri M., Nasiri K. (2023). Gingival mesenchymal stem cell therapy, immune cells, and immunoinflammatory application. Mol. Biol. Rep..

[44] Kim D., Lee A.E., Xu Q., Zhang Q., Le A.D. (2021). Gingiva-Derived Mesenchymal Stem Cells: Potential Application in Tissue Engineering and Regenerative Medicine—A Comprehensive Review. Front. Immunol..

[45] Gao X., Cao Z. (2020). Gingiva-derived Mesenchymal Stem Cells and Their Potential Applications in Oral and Maxillofacial Diseases. Curr. Stem Cell Res. Ther..

[46] Grawish M.E. (2018). Gingival-derived mesenchymal stem cells: An endless resource for regenerative dentistry. World J. Stem Cells.

[47] Fonticoli L., Della Rocca Y., Rajan T.S., Murmura G., Trubiani O., Oliva S., Pizzicannella J., Marconi G.D., Diomede F. (2022). A Narrative Review: Gingival Stem Cells as a Limitless Reservoir for Regenerative Medicine. Int. J. Mol. Sci..

[48] Morsczeck C., Götz W., Schierholz J., Zeilhofer F., Kühn U., Möhl C., Sippel C., Hoffmann K.H. (2005). Isolation of precursor cells (PCs) from human dental follicle of wisdom teeth. Matrix Biol..

[49] Pisciotta A., Carnevale G., Meloni S., Riccio M., De Biasi S., Gibellini L., Ferrari A., Bruzzesi G., De Pol A. (2015). Human dental pulp stem cells (hDPSCs): Isolation, enrichment and comparative differentiation of two sub-populations. BMC Dev. Biol..

[50] Gronthos S., Arthur A., Bartold P.M., Shi S. (2011). A method to isolate and culture expand human dental pulp stem cells. Methods Mol. Biol..

[51] Dominici M., Le Blanc K., Mueller I., Slaper-Cortenbach I., Marini F.C., Krause D.S., Deans R.J., Keating A., Prockop D.J., Horwitz E.M. (2006). Minimal criteria for defining multipotent mesenchymal stromal cells. The International Society for Cellular Therapy position statement. Cytotherapy.

[52] Seo B.M., Miura M., Gronthos S., Bartold P.M., Batouli S., Brahim J., Young M., Robey P.G., Wang C.Y., Shi S. (2004). Investigation of multipotent postnatal stem cells from human periodontal ligament. Lancet.

[53] Jin S.H., Lee J.E., Yun J.H., Kim I., Ko Y., Park J.B. (2015). Isolation and characterization of human mesenchymal stem cells from gingival connective tissue. J. Periodontal Res..

[54] Sonoyama W., Liu Y., Yamaza T., Tuan R.S., Wang S., Shi S., Huang G.T. (2008). Characterization of the apical papilla and its residing stem cells from human immature permanent teeth: A pilot study. J. Endod..

[55] Huang G.T., Sonoyama W., Liu Y., Liu H., Wang S., Shi S. (2008). The hidden treasure in apical papilla: The potential role in pulp/dentin regeneration and bioroot engineering. J. Endod..

[56] Iida H. (2017). Oral Health Interventions during Pregnancy. Dent. Clin. N. Am..

[57] Schröter U., Ziebolz D., Stepan H., Schmalz G. (2022). Oral hygiene and oral health behavior, periodontal complaints and oral health-related quality of life in pregnant women. BMC Oral Health.

[58] Al Agili D.E., Khalaf Z.I. (2023). The role of oral and prenatal healthcare providers in the promotion of oral health for pregnant women. BMC Pregnancy Childbirth.

[59] Popovici D., Crauciuc E., Socolov R., Balan R., Hurjui L., Scripcariu I., Pavaleanu I. (2018). Early Diagnosis and Treatment of Dental Caries in Pregnancy. Maedica.

[60] Liu Y., Zheng Y., Ding G., Fang D., Zhang C., Bartold P.M., Gronthos S., Shi S., Wang S. (2008). Periodontal ligament stem cell-mediated treatment for periodontitis in miniature swine. Stem Cells.

[61] Pringle S., Van Os R., Coppes R.P. (2013). Concise review: Adult salivary gland stem cells and a potential therapy for xerostomia. Stem Cells.

[62] Shinmura Y., Tsuchiya S., Hata K.I., Honda M.J., Qu H.C., Takamoto M., Uno K., Hirose Y., Kamisaki Y., Ide Y. (2008). Human salivary gland stem/progenitor cells remain dormant even after irradiation. Int. J. Mol. Med..

[63] Jing J., Feng J., Yuan Y., Guo T., Lei J., Pei F., Ho T.V., Chai Y. (2022). Spatiotemporal single-cell regulatory atlas reveals neural crest lineage diversification and cellular function during tooth morphogenesis. Nat. Commun..

[64] Trounson A., McDonald C. (2015). Stem Cell Therapies in Clinical Trials: Progress and Challenges. Cell Stem Cell.

[65] Lo B., Parham L. (2009). Ethical issues in stem cell research. Endocr. Rev..

[66] Miran S., Mitsiadis T.A., Pagella P. (2016). Innovative Dental Stem Cell-Based Research Approaches: The Future of Dentistry. Stem Cells Int..

[67] Islam N.A.B., Haque A. (2024). Pregnancy-related dental problems: A review. Heliyon.

[68] Shahbazi M.N., Jedrusik A., Vuoristo S., Recher G., Hupalowska A., Bolton V., Fogarty N.N.M., Campbell A., Devito L., Ilic D. (2016). Self-organization of the human embryo in the absence of maternal tissues. Nat. Cell Biol..

